# Antibiotherapy with and without bone debridement in diabetic foot osteomyelitis: A retrospective cohort study

**DOI:** 10.12669/pjms.301.4266

**Published:** 2014

**Authors:** Asim Ulcay, Ahmet Karakas, Mesut Mutluoglu, Gunalp Uzun, Vedat Turhan, Hakan Ay

**Affiliations:** 1 Asim Ulcay, Department of Infectious Diseases and Clinical Microbiology, Gulhane Military Medical Academy, Haydarpasa Teaching Hospital, Uskudar, Istanbul, Turkey.; 2 Ahmet Karakas, Department of Infectious Diseases and Clinical Microbiology, Gulhane Military Medical Academy and School of Medicine, Etlik, Ankara, Turkey.; 3 Mesut Mutluoglu, Department of Underwater and Hyperbaric Medicine, Gulhane Military Medical Academy, Haydarpasa Teaching Hospital, Uskudar, Istanbul, Turkey.; 4 Gunalp Uzun, Department of Underwater and Hyperbaric Medicine, Gulhane Military Medical Academy, Haydarpasa Teaching Hospital, Uskudar, Istanbul, Turkey.; 5 Vedat Turhan, Department of Infectious Diseases and Clinical Microbiology, Gulhane Military Medical Academy, Haydarpasa Teaching Hospital, Uskudar, Istanbul, Turkey.; 6 Hakan Ay, Department of Underwater and Hyperbaric Medicine, Gulhane Military Medical Academy, Haydarpasa Teaching Hospital, Uskudar, Istanbul, Turkey.

**Keywords:** Amputation, Antibiotics, Diabetic foot infection, Osteomyelitis

## Abstract

***Background and Objective:*** The treatment of diabetic foot osteomyelitis (DFO) is a controversial issue, with disagreement regarding whether the best treatment is surgical or conservative. The purpose of this study was to compare the outcome of patients with DFO who were treated with antibiotherapy alone and those who underwent concurrent minor amputation.

***Methods:*** Hospital records of patients who were diagnosed as having DFO within a 2-year study period were retrospectively reviewed. Patients were divided into two groups: those who received antibiotherapy alone and those who underwent concurrent minor amputation. Groups were compared in terms of duration in hospitalization, antibiotherapy, and wound healing.

***Results:*** Thirty seven patients were included in the study. These comprised patients who received antibiotherapy alone (ABG, n=15) and patients who underwent concurrent minor amputation (AB-MAG, n=22). Hospitalization duration was 37.2 (± 16.2) days in ABG and 52.8 (± 40.2) days in AB-MAG (*p* = 0.166). Mean duration of antibiotherapy was 45.0 (± 21.7) days in ABG and 47.7 (± 19) days in AB-MAG (*p* = 0.689). Wound healing duration was 265.2 (± 132.7) days in ABG and 222.6 (± 85.9) days in AB-MAG (*p* = 0.243). None of the outcome measures were significantly different between ABG and AB-MAG.

***Conclusions:*** Our results have shown similar outcomes for both patient groups who received antibiotherapy alone and who underwent concurrent minor amputations. Considering the small sample sizes in this study, it is important to confirm these results on a larger scale.

## INTRODUCTION

Diabetic foot complications account for the majority of non-traumatic lower extremity amputations across the world.^1^ Considering the characteristics of these amputations, a history of ulcer, usually infected, is almost always the most common finding.^2^ Diabetic foot infections are the most prevalent infection type in diabetic patients.^3^ They constitute almost 20% of the hospitalizations related to diabetes mellitus^4^ and account for the longest hospitalization period among all other complications of diabetes mellitus.^3^

Diabetic foot osteomyelitis (DFO) is probably the most difficult-to-treat complication of diabetes mellitus. We have previously shown that the presence of osteomyelitis increases the hospital length of hospital stay, duration of antibiotic therapy, and time to wound healing.^5^ In the past, bone involvement in diabetic foot infections was considered as an absolute indication for lower extremity amputation. In fact, DFO is still widely regarded as a surgical disease.^6^ Today, however, a growing body of literature suggests successful treatment outcomes based on the use of antibiotherapy alone.^7^

The purpose of this study was to compare treatment outcomes of DFO patients treated with antibiotherapy alone and those who underwent concurrent bone debridement.

## METHODS

Patients’ records were retrieved from our diabetic foot patient database.^5^ Patients hospitalized for DFO within a 2-year period and who had 12-month follow-up data were included in the study. In case of multiple hospitalizations, the first was taken into consideration. The diagnosis of foot infection was made based on the criteria of the International Work Group of Diabetic Foot (IWGDF).^8^ DFO diagnosis was made via biopsy whenever the bone was exposed; when this was not the case, the diagnosis was made by means of physical examination findings, laboratory results and other imaging techniques. Magnetic resonance imaging (MRI) was used in all the cases from which a biopsy could not be taken. MRI was performed with a 1.5 Tesla (Magnetom Vision, Siemens, Erlangen-Germany) device. Cases with hypointense imaging on T1 weighted and TIRM series, hyperintense imaging on T2 weighted and TIRM series, and contrast enhancement after injection were diagnosed as DFO.

Lack of palpable pulse in the dorsalis pedis or tibialis posterior arteries of the foot, or signs indicating impairment of blood flow on Doppler ultrasonography were defined as peripheral arterial disease. Diabetic peripheral neuropathy was defined as the inability of the patient to perceive sensation on at least 1 of 4 plantar sites tested with the 10 g Semmes-Weinstein monofilament.^9^ Ulcers were classified based on the diabetic foot ulcer classification of University of Texas (UT).^9^ All patients were managed in accordance with DIME^10^, which is a well established and widely recognized wound care protocol.

Patients were divided into two groups: those who received antibiotherapy alone and those who underwent concurrent minor amputation. All minor amputations were performed beyond the metatarsal level and comprised surgical procedures undertaken at the bedside. The level of minor amputation was primarily determined by the level of gangrene of the overlying tissue, e.g., if only the distal phalanx was gangrenous then only the distal phalanx with the underlying bone was amputated. Groups were then compared to each other in terms of demographics, wound characteristics, and laboratory markers. Treatment groups were compared for three outcome measures: hospitalization duration, total duration of antibiotherapy and total duration of wound healing. Wound healing was defined as complete epithelialization of the wound and as the absence of any clinical signs of wound infection at the end of the follow-up period.

Statistical analyses were made using SPSS software (SPSS Inc., version 11.0, Chicago, IL, USA). In analyzing discrete data, chi-square calculation was used, and in analyzing continuous data, student *t*-test was used. Statistical significance was set at *p* < 0.05. Ethical approval was obtained from the Institutional Review Board.

## RESULTS

Forty-eight patients diagnosed with DFO were eligible for the study during the two years study period. Eleven patients were excluded from the study for following reasons: not completing 12 months follow-up period (n=3); exitus due to a reason other than diabetic foot infection (n=1), major lower extremity amputation due to rapid disease progression (n=7). Remaining 37 patients were included in the analysis. DFO was diagnosed by bone culture in 17 patients (46%) and by means of a combination of clinical, laboratory, and imaging methods as described in the methodology section in the remaining 20 patients (54%).

**Table-I:** Patient characteristics and laboratory test results

*Variables*	*Antibiotherapy*	*Antibiotherapy + Minor amputation*	*p*
N	15	22	
Age (years)	66 ± 13.8	64.3 ± 8.6	0.642
Male/Female	12/3	15/7	0.421
Diabetes duration (years)	14.9 ± 9.7	18.8 ± 11.4	0.284
HbA1c (%)	8.2 ± 2.2	8.5 ± 2.2	0.735
White blood cell count (x10^3^)	9.9 ± 5.1	10.3 ± 4.3	0.798
C-reactive protein (mg/dl)	40.7 ± 56.5	73.7 ± 74.8	0.157
Erythrocyte sedimentation rate (mm/h)	81 ± 38.4	100.8 ± 39.8	0.141
Hemoglobin (mg/dl)	11.2 ± 1.2	10.5 ± 1.6	0.143
Urea (mg/dl)*	54 ± 21.1 (n=14)*	63.2 ± 30.9 (n=21)*	0.342
Creatinine (mg/dl)*	1.2 ± 0.3 (n=14)*	1.3 ± 0.4 (n=21)*	0.504

Data are presented as n (%) or as mean ± SD. HbA1c, glycosylated hemoglobin * Dialysis patients excluded.

**Table-II:** Wound characteristics of patients

*Variables*		*Antibiotherapy*	*Antibiotherapy + Minor amputation*	*p*
n		15	22		
*Etiology*					
	Neuropathic	6 (40)	1 (4.5)	0.004
	Ischemic	1 (6.7)	-	
	Neuro-ischemic	8 (53.3)	21 (95.5)	
*Classification of Texas University*				
	Grade 3 stage B	5 (33.3)	1 (4.5)	0.031
	Grade 3 stage D	10 (66.7)	21 (95.5)	
*PEDIS infection classification*				
	Grade 1	-	-	0.041
	Grade 2	9 (60)	6 (27.3)	
	Grade 3	6 (40)	15 (68.2)	
	Grade 4	-	1 (4.5)	
*Ulcer location *				
	Great toe	6 (40)	6 (27.3)	0.432
	Little toes	3 (20)	9 (40.9)	
	Metatarsus	3 (20)	7 (31.8)	
	Middle of the foot	1 (6.7)	-	
	Heel	2 (13.3)	-	

Data are presented as n (%).

**Table-III:** Comparison of patients who underwent minor amputation and patients who did not in terms of treatment results

	*Antibiotherapy*	*Antibiotherapy + Minor amputation*	*p*
Hospitalization duration (days)	37.2± 16.2	52.8 ± 40.2	0.166
Total duration of antibiotherapy (days)	45.0 ± 21.7	47.7 ± 19	0.689
Wound healing duration (days)	265.2 ± 132.7	222.6 ± 85.9	0.243

Data are presented as mean ± SD.

Patients were divided into two groups: those who received antibiotherapy alone (ABG, n=15) and those who underwent concurrent minor amputation (AB-MAG, n=22). Nine patients in AB-MAG were admitted to our center due to postoperative wounds. Patient characteristics and laboratory test results are presented on Table-I. Mean age of the patients (±SD) was 66 (± 13.8) years in ABG and 64.3 (±8.6) years in AB-MAG (*p* = 0.642); mean diabetes duration was 14.9 (± 9.7) years in ABG and 18.8 (± 11.4) years in AB-MAG (*p* = 0.284), and mean HbA1c levels were 8.2 (±2.2) % in ABG and 8.5 (±2.2) % in AB-MAG (*p* = 0.735). Treatment groups were similar in terms of age, sex, diabetes duration and HbA1c level Table I. Infection markers and renal functions were also similar in ABG and AB-MAG. Wound characteristics of patients are presented on Table-II.

During the 12-month follow-up, recurrence was observed in three patients in each group (*p* = 0.669). None of the outcome factors were significantly different between ABG and AB-MAG. Hospitalization duration was 37.2 (± 16.2) days in ABG and 52.8 (± 40.2) days in AB-MAG (*p* = 0.166). Mean duration of antibiotherapy was 45.0 (± 21.7) days in ABG and 47.7 (± 19) days in AB-MAG (*p* = 0.689). Wound healing duration was 265.2 (± 132.7) days in ABG and 222.6 (± 85.9) days in AB-MAG (*p* = 0.243) Table-III.

## DISCUSSION

The treatment of DFO is challenging. Impaired perfusion of the foot in diabetic patients renders the underlying bones susceptible to infection and diminishes the efficacy of antibiotherapy.^11^ Recurrence is very common and chronicity is an extreme challenge in this particular group of diabetic patients. The orthodox view, commonly shared among surgeons, supports the early surgical excision of all infected bone, either necrotic or not, to eradicate osteomyelitis more successfully and permanently.^6^ Today however, a growing body of literature suggests successful treatment outcomes based on the use of antibiotherapy alone.^12^^-^^18^

Game and Jeffcoate reviewed their records of patients presenting with diabetic foot osteomyelitis and identified 147 patients, 26 (18%) of whom were hospitalized and delivered parenteral antibiotics. While 113 of their patients received antibiotherapy alone, 34 (23%) underwent major (6/34) or minor (28/34) amputations. They reported similar remission rates in both the surgical and non-surgical groups (78.6%, 83.3%, respectively).^12^ Senneville et al^13^ in a multi-center study with 50 consecutive patients diagnosed with DFO and managed with antibiotherapy alone, reported remission in 32 (64%) patients. Valabhji et al^14^ achieved a 83% remission rate in 47 patients diagnosed with DFO and managed non-surgically. Our results are in line with several other studies.^15^^-^^18^

We found similar outcomes between patients who received antibiotherapy alone and those who underwent concurrent minor amputation. The high rate in remission rates at the end of 12 months in both groups may be attributed to the fact that antibiotherapy durations were equal to or more than the contemporary treatment guidelines on one hand, and to the application of an extensive and aggressive wound care protocol depending on bone debridement or contemporary wound treatment principles on the other. In other respects, the fact that both antibiotherapy and hospitalization duration, although not significant, are longer in AB-MAG can be relevant to the fact that patients in this group were admitted with more severe wounds in the first place. Likewise, all patients, except for one, were classified under UT grade: 3 stage:D (95.5%) in AB-MAG while this percentage was lower in ABG (66.6%).

Similarly, according to the clinical severity classification of PEDIS infections, in AB-MAG infection rate with erythema less than 2 cm was 27.3% while infection rate with erythema more than 2 cm was 68.2%; cases in which toxicity was involved were 4.5%. But in ABG, infection rate with erythema less than 2 cm was 60% while infection rate with erythema more than 2 cm was 40%; and there were no cases in which toxicity was involved. Subject to all these factors, longer antibiotherapy and hospitalization duration in AB-MAG can be considered as normal even though minor amputation was performed.

When reviewing the post-treatment recovery period, it is seen that patients in AB-MAG healed in shorter periods, although this was not statistically significant (222.6±85.9 days vs 265.2± 132.7, *p* = 0.243). This difference may point to the positive effect of bone debridement on the treatment process. Yet another detail that makes this difference more significant is that 9 of the AB-MAG patients were admitted with non-healing postoperative wound complications, and therefore they were considered as more difficult cases.

Besides its retrospective nature, this study has several other limitations. The likely reason why none of our findings reached the level of statistical significance may be that there were few cases and thus the study was underpowered. Even though the fact that the diagnosis was not made with biopsy in all patients, bone biopsies cannot be commonly performed because they are an invasive application and require surgical experience and skills. Even though it is not considered the gold standard in diagnosis of osteomyelitis, MRI is a diagnostic method, which has high sensitivity and specificity for DFO if it is interpreted by an experienced radiologist.^19^^-^^22^

## CONCLUSIONS

Our results have shown similar outcomes between patients who received antibiotherapy alone and those who underwent concurrent minor amputations. These results, however, should be interpreted in the light of the fact that wounds in the AB-MAG group were more severe, perhaps as a result of selection bias. Confirming these findings with a more prospective and randomized study with sufficient power could contribute to the discussion of optimal DFO treatment significantly.

## Authors Contributions:

AU: conceived, designed the protocol & prepared the final manuscript.

AK: contributed in manuscript writing.

MM, GU: collected patients records and manuscript writing. 

VT: was involved in clinical management of patients.

HA : did review and final approval of manuscript.
